# Choreographing the motor-driven endosomal dance

**DOI:** 10.1242/jcs.259689

**Published:** 2022-11-16

**Authors:** Marlieke L. M. Jongsma, Nina Bakker, Jacques Neefjes

**Affiliations:** Department of Cell and Chemical Biology, ONCODE institute, Leiden University Medical Center LUMC, 2333 ZC Leiden, The Netherlands

**Keywords:** Dynein, Dynactin, Kinesin, Microtubules, Endosomes, Endoplasmic reticulum, Membrane contact sites

## Abstract

The endosomal system orchestrates the transport of lipids, proteins and nutrients across the entire cell. Along their journey, endosomes mature, change shape via fusion and fission, and communicate with other organelles. This intriguing endosomal choreography, which includes bidirectional and stop-and-go motions, is coordinated by the microtubule-based motor proteins dynein and kinesin. These motors bridge various endosomal subtypes to the microtubule tracks thanks to their cargo-binding domain interacting with endosome-associated proteins, and their motor domain interacting with microtubules and associated proteins. Together, these interactions determine the mobility of different endosomal structures. In this Review, we provide a comprehensive overview of the factors regulating the different interactions to tune the fascinating dance of endosomes along microtubules.

## Teaser

**Summary:** Endosomal transport requires dynein and kinesin motors to associate with the endosomal membrane and the microtubule. We summarize the factors regulating these different interactions thereby tuning the fascinating endosomal dance.

## Introduction

**Fig. 1.**
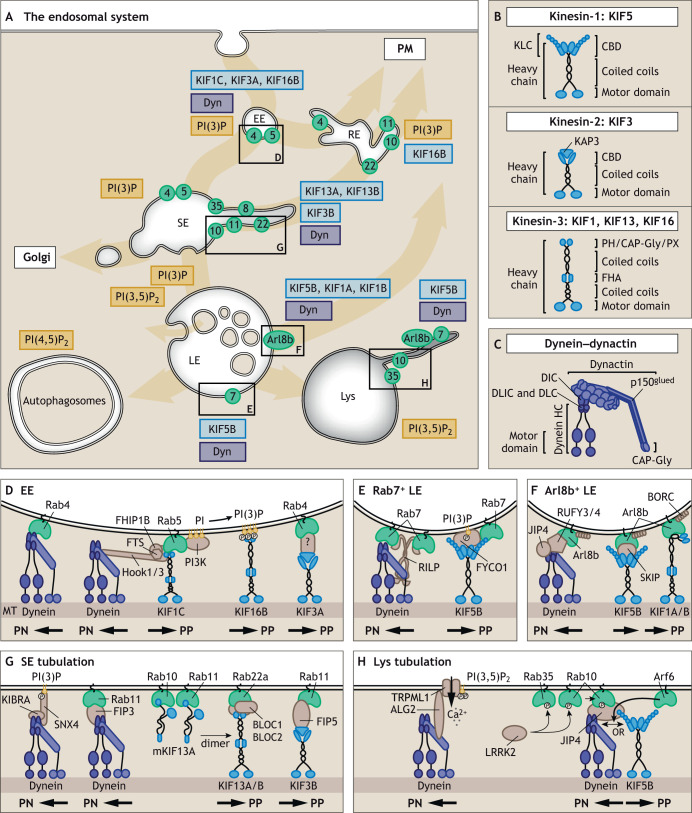
**Small GTPases, PIPs and effector proteins at the endosomal membrane recruit dynein and kinesin motors.** (A) Overview of the endosomal system. Early endosomes (EE), sorting endosomes (SE), recycling endosomes (RE), late endosomes (LE), lysosomes (Lys) and autophagosomes are shown. Each compartmental membrane is marked with a unique set of GTPases (green circles with indicated Rab number) and PIPs (yellow) that recruit kinesin (light blue) and/or dynein motors (dark blue). (B,C) Schematics of kinesin-1, kinesin-2 and kinesin-3 complexes (B) and dynein–dynactin (C). Cofactors and domains of the motor heavy chains are indicated. (D–H) Schematics of the GTPases (green), PIPs (yellow), effector proteins (brown) and motor proteins (light and dark blue) recruited to distinct endosomal membranes. (D) Motor proteins are recruited to the EE membrane through interactions with the small GTPases Rab4 and Rab5, and PI(3)P. (E) Motor proteins are recruited to Rab7^+^ LEs through interactions with the small GTPase Rab7 and PI(3)P. (F) Motor proteins are recruited to Arl8b^+^ LEs through interactions with the small GTPase Arl8b. (G) Tubulation at the SE membrane requires motor protein recruitment through the small GTPases Rab10, Rab11 and Rab22a, and PI(3)P. Rab10 and Rab11 recruit KIF13A monomers, whereas Rab22a dimerizes and activates KIF13A. (H) Tubulation at the Lys membrane requires motor protein (light and dark blue) recruitment through the small GTPases Rab35 and Rab10, and PI(3,5)P_2_. The kinase LRRK2 phosphorylates Rab35 and Rab10 followed by JIP4 binding to Rab10. JIP4 can interact with both dynein and kinesin, which is controlled by Arf6. PI(3,5)P_2_ stimulates Ca^2+^ release through the TRPML1 Ca^2+^ channel, thereby activating the Ca^2+^ sensor ALG2 which recruits dynein. MT, microtubule.

In mammalian cells, different types of endosomes form a highly organized continuous system, the endosomal system, which is essential for the maintenance of cellular homeostasis (O'Sullivan and Lindsay, 2020; Lim and Zoncu, 2016). The various members of the endosomal pathway can be defined as early endosomes (EEs), sorting endosomes (SEs), recycling endosomes (REs), late endosomes (LEs) and lysosomes (Lys) (Fig. 1A). These are highly dynamic structures that mature, move, fuse and communicate with other organelles to enable the organized transport of lipids, proteins and nutrients in the cellular space (Lim and Zoncu, 2016). Endocytosed material first enters EEs, which mature into SEs (Cullen and Steinberg, 2018). While some proteins enter tubular structures for recycling to the plasma membrane or trans-Golgi network, the SEs mature into LEs characterized by decreased luminal pH and intraluminal vesicles (ILVs) (Cullen and Steinberg, 2018). A fraction of the ILVs will fuse back to the limiting membrane of the LEs through a process termed retrofusion, whereas other ILVs are released as exosomes when LEs fuse with the plasma membrane (Perrin et al., 2021). Other LEs mature and fuse with lysosomes for final proteolytic degradation of their content (Bright et al., 2016; Edgar, 2016) (Fig. 1A). Notably, the intracellular localization of LEs determines their fate; those destined for exosome secretion are transported towards the plasma membrane whereas fusion of LEs with lysosomes occurs at the perinuclear area. Endosomal localization can be adjusted to cellular needs. For example, during starvation, lysosomes are directed towards autophagosomes at the perinuclear area, where they fuse to form autolysosomes, providing the acidic environment required to free nutrients (Lorincz and Juhasz, 2020).

The identity of each endosomal subtype is determined by its membrane composition, in particular the presence of specific small GTPases of the Rab and Arf/Arl family and their interacting effector proteins, along with the membrane lipid composition, which mainly differs in phosphatidylinositol phosphate (PIP) species between endosomal subtypes (Stenmark, 2009; Donaldson and Honda, 2005; Balla, 2013; Li and Marlin, 2015; Posor et al., 2022) (Fig. 1A). This endosomal identity is essential for controlled recruitment of motor proteins, which are the driving forces behind endosomal trafficking and ensure endosomal fate. Long-range endosomal transport along microtubules is bidirectional and regulated by the alternating activities of kinesin and dynein motors, which transport endosomes to the microtubule plus-end (anterograde transport) and minus-end (retrograde transport), respectively (Endow et al., 2010; Hook and Vallee, 2006). A third motor family, myosins, regulate short-distance endosomal transport along actin filaments using various mechanisms; these have been recently reviewed (Bonifacino and Neefjes, 2017).

The kinesin superfamily (or KIFs) is highly diverse, with 14 subfamilies, covering a total of 45 different kinesin heavy chains (Lawrence et al., 2004; Hirokawa et al., 2009). These heavy chains form dimers through their coiled-coil domains, and form complexes with cofactors to assemble into different kinesin variants for transport of a wide variety of cargoes, including different endosomes, other organelles and mRNA (Verhey and Hammond, 2009; Dimitrova-Paternoga et al., 2021). The general architecture of kinesin motors features a C-terminal cargo-binding domain (CBD) and an N-terminal motor domain for endosomal plus-end-directed transport (Fig. 1B). There are two exceptions to this general principle – members of the kinesin-14 family have switched the position of the motor domain (now C-terminal), thereby facilitating endosomal movement in the opposite minus-end direction (Cross, 2010), and the kinesin-13 family members have their motor domain in the middle, which precludes them from transporting endosomes and instead they depolymerize microtubules, leading to shorter microtubule tracks (Ogawa et al., 2004; Chatterjee et al., 2016). In contrast to the large number of available kinesin heavy chains, mammalian cells express only one cytoplasmic dynein heavy chain (DHC) for minus-end transport of a large variety of cargoes, including all endosomal vesicles (Vallee et al., 2004). Specificity in cargo binding and functional diversity is attained by complex formation between DHC dimers and various dynein intermediate chains (DICs), dynein light-intermediate chains (DLICs), dynein light chains (DLCs), and the multi-subunit cofactor dynactin (Deacon et al., 2003; King et al., 2003; Schroer, 2004) (Fig. 1C). The dynactin subunit p150^glued^ (also known as DCTN1) supports and stabilizes the interaction between dynein and the microtubule through interaction with DICs (King et al., 2003; Vaughan and Vallee, 1995), whereas the p150^glued^ CAP-Gly-domain interacts with the microtubule (Bjelic et al., 2012) (Fig. 1C).

Optimal functioning and positioning of the endosomal system requires a perfect balance between kinesin- and dynein-mediated transport. Not surprisingly, disordered endosomal transport has been implicated in many diseases, including lysosomal storage diseases (LSDs) such as Niemann–Pick disease, cancer and neurodegenerative diseases (Ballabio and Bonifacino, 2020). For example, in Niemann–Pick disease, a mutation in the cholesterol transporter NPC1 or NPC2 results in cholesterol accumulation in lysosomes. This excess cholesterol is sensed by a protein called ORP1L (also known as OSBPL1A) to allow dynein-mediated transport and lysosome accumulation in the perinuclear area, affecting the proteolytic function of lysosomes (Rocha et al., 2009). Moreover, some cancer types enhance the fusion of lysosomes and autophagosomes to generate sufficient nutrients for fast cancer cell growth (Kimmelman and White, 2017). These examples illustrate the importance of accurate endosomal transport.

In this Review, we describe how kinesin and dynein motors coordinate endosomal transport – how they are recruited to specific endosomal membranes and microtubules in a controlled manner, and how endosomal transport is regulated by other cellular compartments, most notably the endoplasmic reticulum (ER).

## Motor protein recruitment to the endosomal membrane

A first and critical step for endosomal transport is that motor proteins detect and interact with specific types of endosomes. Motor protein binding to the endosomal membrane requires interactions with endosomal anchors, the small GTPases and PIPs. The small GTPases cycle between an active GTP-bound membrane-localized and an inactive GDP-bound cytosolic state. These states are regulated by guanine nucleotide exchange factors (GEFs) and GTPase-activating proteins (GAPs), respectively (Homma et al., 2021). In their active state, small GTPases recruit specific effector proteins and cofactors, providing the binding platform for kinesin and/or­ dynein motors. Motor recruitment is further supported by PIPs, which are binding partners for motor proteins and effector proteins that contain lipid-binding-domains, such as PH, PX and FYVE domains. The present small GTPases and PIPs give each endosomal subtype a unique identity. For example, EEs are mostly marked by Rab5 (collectively referring to the Rab5a, Rab5b and Rab5c isoforms unless otherwise specified) and PI(3)P, SEs by Rab11 (Rab11a and Rab11b) and PI(3)P, and LEs by Rab7 (Rab7a and Rab7b) and PI(3,5)P_2_ (Fig. 1A). This identity can be recognized by a specific set of motor proteins allowing variation in transport characteristics between endosomal subtypes during endosomal maturation as well as during endosomal tubulation.

### Motor protein recruitment during endosomal maturation

Given that the endosomal system is continuous, the different endosomal subtypes mature from one subtype to the other by subsequently changing the GTPase and lipid composition of their membranes, a process called endosomal maturation. The maturation of EEs into LEs has been extensively studied and is understood in great detail. During this process, EEs undergo a Rab5-to-Rab7 identity switch (Rink et al., 2005). Rab5 recruits the C18orf8–Mon1–Ccz1 complex (C18orf8 is also known as RMC1, and there are Mon1a and Mon1b isoforms in mammals), which is the GEF for Rab7 that brings Rab7-GTP onto the membrane (Nordmann et al., 2010; Poteryaev et al., 2010; van den Boomen et al., 2020). Subsequently, Rab7 recruits the Rab5 GAP TBC1D2, thereby clearing Rab5 from the now Rab7-marked LE membrane (Chotard et al., 2010). This handover mechanism for endosome identity continues when a subset of Rab7^+^ LEs replace Rab7 with another small GTPase, Arl8b. The switch is regulated by the Arl8b effector SKIP (also known as PLEKHM2), which forms a bridge between Rab7 and Arl8b. This is followed by HOPS (‘homotypic fusion and protein sorting’) complex recruitment and subsequent binding of TBC1D15, a GAP for Rab7, which clears Rab7 from the membrane to yield an endosome marked by Arl8b only (Jongsma et al., 2020). Rab switching is coupled to changes in lipid composition. At the EE membrane, Rab5 recruits the phosphoinositide 3-kinase (PI3K) complex (Beclin-1–Vps15–Vps34; Vps15 and Vps34 are also known as PIK3R4 and PIK3C3, respectively), which phosphorylates PI into PI(3)P (Christoforidis et al., 1999; Murray et al., 2002) (Fig. 1D). When EEs mature, PI(3)P recruits the kinase PIKFYVE as well as myotubularin (MTM) family members to convert PI(3)P into PI(3,5)P_2_ (Ikonomov et al., 2001; Robinson and Dixon, 2006). The unique identity of each endosomal subtype can recruit different types of motor proteins, which is discussed below in detail for some of the small GTPases and lipids present on EEs, Rab7^+^ LEs and Arl8b^+^ LEs (Fig. 1D–F; Table S1).

The EE membrane is mostly decorated with Rab4 (Rab4a and Rab4b), Rab5 and PI(3)P, which all recruit different motor proteins that in assembly orchestrate EE movement. Rab5 interacts with the FHF adaptor complex (FHIP1B–Hook1, 2 or 3–FTS; FTS is also known as AKTIP), forming a docking platform at the EE membrane for both dynein and the kinesin-3 motor KIF1C (Bielska et al., 2014; Christensen et al., 2021; Guo et al., 2016; Kendrick et al., 2019; Schroeder and Vale, 2016; Yao et al., 2014; Zhang et al., 2014) (Fig. 1D). Rab4 can bind either directly or indirectly to kinesin-2 (KIF3A) in neuronal cells (Dey et al., 2017), and directly to the dynein subunit DLIC-1 in *Drosophila* (Bielli et al., 2001) (Fig. 1D). PI(3)P recruits kinesin-3 (KIF16B) through binding to the kinesin PX domain (Blatner et al., 2007; Hoepfner et al., 2005; Pyrpassopoulos et al., 2017) (Fig. 1D). Together, this suggests a coordinated action of dynein and the kinesins KIF1C, KIF3A and KIF16B for functional EE transport but how the different motors act in time and space is unclear. LE membranes are mostly marked by Rab7, Arl8b, PI(3)P and PI(3,5)P_2_. Rab7 has at least two different effector proteins, RILP and FYCO1, which recruit dynein and kinesin motors, respectively. If Rab7 binds RILP, the dynein motor is recruited through direct binding to the C-terminus of the dynactin subunit p150^glued^ for retrograde transport (Jordens et al., 2001; Johansson et al., 2007) (Fig. 1E). When Rab7 and PI(3)P recruit the FYVE-domain-containing effector FYCO1 (Pankiv et al., 2010), kinesin-1 (KIF5B) motors are recruited to the LE membrane, shifting the balance towards anterograde movement (Pankiv et al., 2010) (Fig. 1E). After further maturation, Rab7 is released and Arl8b is recruited to the LE membrane by BORC [biogenesis of lysosome-related organelles complex 1 (BLOC-1) related complex] (Pu et al., 2015; Jongsma et al., 2020). Arl8b mediates anterograde transport by direct binding to the kinesin-3 (KIF1A and/or KIF1B) CC3 domain (Guardia et al., 2016) or kinesin-1 (KIF5) through its effector protein SKIP (Pu et al., 2015; Rosa-Ferreira and Munro, 2011) (Fig. 1F). In contrast, retrograde transport of Arl8b^+^ LEs is regulated by the Arl8b effectors RUFY3 and RUFY4, which recruit JIP4 (also known as SPAG9) and the dynein complex (Keren-Kaplan et al., 2022; Kumar et al., 2022) (Fig. 1F). Overall, the presence of multiple GTPases, lipids and effector protein at EEs and LEs allows them to interact with both dynein and different kinesin proteins resulting in bidirectional movement.

### Motor protein recruitment during endosomal tubulation

SEs are the main sorting stations of the endosomal system where cargo that is not destined for degradation is directed into tubular structures for recycling to the plasma membrane or Golgi. Force needed for tubulation can be generated by the kinesin-3 motor KIF13A (Roux et al., 2002; Thankachan and Setty, 2022). Inactive KIF13A monomers are recruited to SE budding regions by Rab10 and Rab11 (Delevoye et al., 2014; Etoh and Fukuda, 2019), whereafter Rab22a interacts with the multisubunit complexes BLOC-1 (BLOC1S1–6, DTNBP1 and SNAPIN) and BLOC-2 (HPS3, HPS5 and HPS6), and the KIF13A CC1 domain, releasing motor inhibition and allowing formation of active KIF13A dimers (Shakya et al., 2018) (Fig. 1G). Kinesin-driven transport leads to extension of the buds into tubules (Etoh and Fukuda, 2019; Patel et al., 2021; Shakya et al., 2018; Soppina et al., 2014). Another kinesin described to play a role in tubulation at the SE is the kinesin-2 KIF3B, which binds the tubule through an interaction with the Rab11-binding protein Rip11 (also known as RAB11FIP5) (Schonteich et al., 2008) (Fig. 1G). In addition, SEs recruit dynein through Rab11 and its effector FIP3 (RAB11FIP3), or through KIBRA (WWC1) and SNX4 of which the latter binds PI(3)P at the SE membrane via its PX domain (Skanland et al., 2009; Traer et al., 2007; Horgan et al., 2010) (Fig. 1G). When tubules have been formed, Rab8a recruits EHBP1L1, Bin-1 and dynamin for fission (Nakajo et al., 2016). Rab8 is recruited to the SE tubules when MICAL-L1 is present (Sharma et al., 2009), which is controlled by Arf6 and Rab35. Although Arf6 stimulates MICAL-L1 membrane localization, Rab35 inhibits tubular localization of MICAL-L1 directly (Rahajeng et al., 2012) as well as by inactivating Arf6 through the recruitment of the Arf6 GAP ACAP2 (Kanno et al., 2010). Tubulation also occurs at the LE and Lys membrane, and requires coordinated kinesin and dynein recruitment (Li et al., 2016; Mrakovic et al., 2012). In macrophages, LE and Lys tubulation involves the GTPases Rab7 and Arl8b and the effector proteins for kinesin and dynein recruitment as described above for LEs (Mrakovic et al., 2012) (Fig. 1E,F). In addition, LE and Lys tubulation can be regulated by PI(3,5)P_2_ and the kinase LRRK2. PI(3,5)P_2_ at the lysosomal membrane triggers the opening of the lysosomal Ca^2+^ channel TRPML1 (also known as MCOLN1) leading to increased cytosolic Ca^2+^ levels, which activates the dynein binding lysosomal Ca^2+^-sensor ALG2 (Li et al., 2016) (Fig. 1H). LRRK2 phosphorylates Rab35 and Rab10 which supports JIP4 recruitment to Rab10 at tubular structures and subsequent JIP4 motor binding (Bonet-Ponce et al., 2020) (Fig. 1H). Although JIP4 is mainly reported to interact with dynein, a study on endosomal transport during mitosis identified JIP4 as a kinesin-1- and dynein-binding protein controlled by Arf6-GTP (Montagnac et al., 2009) (Fig. 1H). By recruiting kinesin and dynein motors, opposite forces can be created supporting the formation of tubular structures, yet it is only poorly understood how kinesin and dynein motors cooperate inside cells.

## Cellular signals that control motor recruitment to endosomal membranes

Dynein and kinesin motors are present in excess over the number of small GTPases and lipid anchors at the endosomal membrane, allowing rapid recruitment in response to cellular needs. Most motor proteins are inactive, reside in the cytosol (and possibly the endosomal membrane) and require activation before bringing endosomes into motion. To become active, motor proteins have to switch from an auto-inhibited to an active conformation (Zhang et al., 2017; Coy et al., 1999; Cai et al., 2007; Friedman and Vale, 1999). Kinesin motors undergo this conformational change when their tail region interacts with small GTPases, adaptors and lipids at the endosomal membrane. Auto-inhibition of dynein motors is released upon binding to its cofactor, the dynactin complex, and further stabilized by activating endosomal adaptors, such as BICD2, FIP3 and Hook3 (Hoogenraad and Akhmanova, 2016; Horgan et al., 2010; Reck-Peterson et al., 2018; Schroeder and Vale, 2016). The activation and recruitment of motor proteins to the endosomal membrane is further controlled by signaling cues, including Rab GTPase activity, post-translational modifications (PTMs), lipids and alterations in Ca^2+^ levels, adding an additional level of control to individual vesicle movement (Fu and Holzbaur, 2014; Schlager and Hoogenraad, 2009; Akhmanova and Hammer, 2010; Hoogenraad and Akhmanova, 2016).

### Cells adjust endosomal transport in response to cellular cues such as starvation

To generate nutrients during starvation, lysosomes and autophagosomes are redistributed to the perinuclear area where they efficiently fuse (supported by the HOPS complex) to recycle the autophagosomal contents for cellular needs (Korolchuk et al., 2011; Khobrekar and Vallee, 2020; van der Kant et al., 2013). Lysosome clustering upon starvation follows TFEB–TFE3-mediated TMEM55B (also known as PIP4P1) upregulation, enhancing TMEM55B–JIP4–dynein-mediated retrograde transport. Also, PI(3,5)P_2_ is generated at the lysosomal membrane and triggers TRPML1–ALG2–dynein transport as discussed above for LE and Lys tubulation (Willett et al., 2017; Li et al., 2016; Ikari et al., 2020; Dong et al., 2010) (Fig. 1H). Retrograde autophagosome transport depends on an interaction between RILP and autophagosomal LC3B (also known as MAP1LC3B), which recruits dynein as shown in neurons (Khobrekar and Vallee, 2020). Meanwhile, Arl8b-mediated anterograde transport is inhibited during limited nutrient availability. Although in normal conditions Arl8b is recruited to the LE and Lys membrane by BORC, during nutrient starvation BORC interacts with a protein called Ragulator (a protein complex involved in mTORC1 signaling), which limits Arl8b recruitment (Filipek et al., 2017; Pu et al., 2017). In summary, starvation signals induce a change in small GTPase recruitment, lipid composition and Ca^2+^ release, shifting the balance towards retrograde transport mechanisms of lysosomes and autophagosomes. This brings both endosomal compartments in close proximity, which accelerates the downstream fusion processes and subsequent nutrient release.

## Kinesin and dynein motors coordinate their activity for efficient transport

**Fig. 2.**
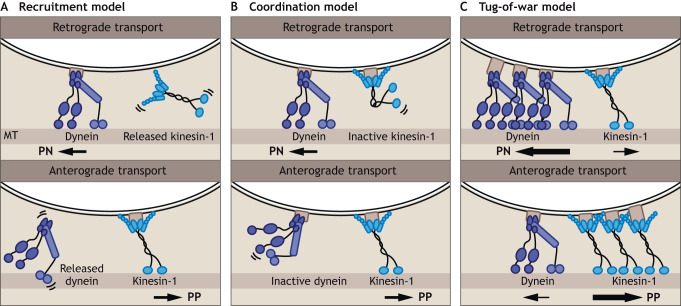
**Three models for bidirectional endosomal movement.** (A) In the recruitment model, dynein and kinesin motors alternate their recruitment to the endosomal membrane. Directionality is determined by the type of motor bound at a defined moment in time. (B) In the coordination model, both dynein and kinesin motors are associated with the endosome, but only one type of motor is active at a given time. (C) In the tug-of-war model, both motors are actively bound to the endosome, providing force in opposite directions. The team of motors generating the greatest force wins and determines the direction of movement. Kinesin-1 is shown as an example kinesin; PN, perinuclear; PP, periphery.

To allow efficient bidirectional endosomal transport and tubulation, endosomal transport depends on a coordinated balance between kinesin- and dynein-mediated movements. Given that the endosomal membrane contains multiple motor anchors, it is likely that multiple, even oppositely directed, motor proteins bind one individual endosome simultaneously. How do endosomes move with multiple motors attached? Three models have been proposed (Fig. 2). In the recruitment model, motor proteins associate and dissociate constantly from the endosomal membrane and directionality is determined by the type of motor bound at a defined moment in time (Fig. 2A). However, the biological observation that co-existence of dynein and different kinesin motors on one individual endosomal membrane are essential for proper transport does not support this model (Grigoriev et al., 2007). Another model, the coordination model states that kinesin and dynein motors are continuously bound to their cargo, but only one type of motor is active at a time (Reis et al., 2012) (Fig. 2B). Motor activity is regulated by activating effector proteins or PTMs, which allows rapid directional changes (Maday et al., 2012; Zajac et al., 2013). In support of this model, the kinesin-3 motor KIF1C and dynein co-exist in a complex with the dynein-activating adaptor Hook3 on early endosomes (Fig. 1D), allowing movement towards both the microtubule plus-end and minus-end by coordinated activation of both motors. A third model, the tug-of-war model, proposes that both motors are actively bound to the cargo and exert opposing forces along the microtubule. The team of motors generating the greatest force wins and determines directionality, while the opposing motors then detach from the microtubule, preventing crowding of the tracks (Fig. 2C). This is supported by advanced *in vitro* experiments where motor proteins have been attached to beads (Belyy et al., 2016). It is currently unclear which model best explains bidirectional endosomal movement inside the cell. Many experiments are performed *in vitro*, studying biophysical motor properties under artificial conditions, excluding additional cellular factors (known and unknown), which might not reflect the *in vivo* situation.

## Motor proteins bind microtubule tracks to transport endosomes

To transport endosomes, motor proteins connect to microtubular highways, which cover the entire cytosol to ensure endosomal transport to many cellular destinations. Motor protein binding and their initiation of movement along the microtubule tracks is controlled by microtubule-associated proteins (MAPs) bound to the microtubule surface and by the characteristics of tubulin subunits, which are the microtubule building blocks.

### MAPs control endosomal transport along microtubules

**Fig. 3.**
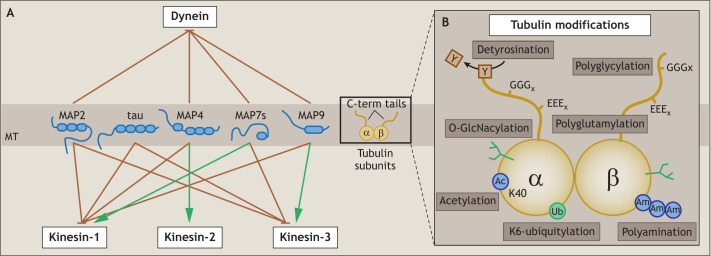
**MAPs and tubulin-PTMs affect motor binding to the microtubule.** (A) Selection of MAPs and their inhibiting (orange lines) or stimulating (green lines) effects on dynein, kinesin-1, kinesin-2 and kinesin-3 motility. (B) PTMs that can be present on the α- and β-tubulin subunits of the microtubule.

Endosomal transport and localization requires a defined architectural and dynamically balanced microtubule network (Matteoni and Kreis, 1987). The formation of this network depends on MAPs, which play a role in microtubule structure, stability, organization and dynamics (Goodson and Jonasson, 2018; Jijumon et al., 2022; Bodakuntla et al., 2019). Some other MAPs are known to alter dynein and/or kinesin motility (Fig. 3A; Table S2). This was first shown for MAP2, which binds to the same spot on the microtubule as dynein, kinesin-1 and kinesin-3, thereby preventing motor binding and inhibiting both retrograde and anterograde endosomal transport (Paschal et al., 1989; Hagiwara et al., 1994; Monroy et al., 2020) (Fig. 3A). Structurally related to MAP2 is the neuronal MAP tau (Dehmelt and Halpain, 2005). Like MAP2, tau inhibits kinesin-1 and kinesin-3-mediated endosomal transport but does not affect dynein and kinesin-2 motility (Chaudhary et al., 2018; Dixit et al., 2008; Monroy et al., 2018; 2020) (Fig. 3A). Interestingly, EE transport (mostly controlled by kinesin-3 and kinesin-2) is more affected by tau binding than LE transport (mostly regulated by kinesin-1 and kinesin-2) (Balabanian et al., 2017) (Fig. 1D–F). This difference might be the result of a phosphorylated form of tau that inhibits kinesin-3 function, but not kinesin-1, even more strongly than the non-phosphorylated form (Balabanian et al., 2022). By inhibiting kinesin motors, tau favors retrograde endosomal movement (Chaudhary et al., 2018). By contrast, other MAPs, including MAP4, MAP7 and MAP9, disturb dynein-mediated endosomal transport while promoting kinesin-mediated anterograde endosomal movement (Ferro et al., 2022; Semenova et al., 2014; Lopez and Sheetz, 1993; Paschal et al., 1989) (Fig. 3A). For example, MAP9 inhibits the dynein complex by blocking the interaction between microtubules and the dynactin subunit p150^glued^, but enhances kinesin-3 motility (Monroy et al., 2020). MAP4 stimulates kinesin-2 motility (Semenova et al., 2014), and MAP7 family members promote kinesin-1 recruitment to the microtubule as well as kinesin-1 activation (Barlan et al., 2013; Chaudhary et al., 2019; Hooikaas et al., 2019; Monroy et al., 2018; Song et al., 2013) (Fig. 3A). The MAP7 family consists of MAP7, MAP7D1, MAP7D2 and MAP7D3, which have redundant functions in enhancing kinesin-1 motility, but differ in their cellular location and mechanism of action, as recently determined for MAP7 and MAP7D3 (Hooikaas et al., 2019). Whereas MAP7 is ‘stuck’ at perinuclear microtubules (Serra-Marques et al., 2020), allowing kinesin-1 to ‘hop’ from one MAP7 to the next, MAP7D3 is more dynamic and moves together with kinesin-1 along microtubules towards the periphery (Hooikaas et al., 2019). The diversity in MAPs gives microtubules unique spatial and timely control of specific motor protein binding and thus coordination of endosomal transport. It is currently unclear how cells control and adjust MAP patterning within the different cellular regions. In addition, enzymes and modifications, such as phosphorylation (Semenova et al., 2014), might control MAPs and this could provide an additional level of microtubule-based endosomal transport regulation.

### Tubulin subunits and tubulin-PTMs control endosomal transport

Microtubules are polymers of α-tubulin and β-tubulin dimers; the dimers are composed of a mixture of nine different α-tubulin and nine different β-tubulin isotypes. Although incorporation of the different tubulin subunits only mildly changes the mechanical and dynamic microtubule properties (Joshi and Cleveland, 1990; Lewis et al., 1987; Luduena, 1993), their exposed C-terminal tails differ and can be decorated with varying PTMs. Some tubulin-PTMs, such as polyglycylation, polyamination, O-GlcNacylation and ubiquitylation affect microtubule dynamics and stability (Bosch Grau et al., 2013; Song et al., 2013; Ji et al., 2011; Mukherjee et al., 2017). Other PTMs, such as polyglutamylation, acetylation and detyrosination, sequentially modulate MAP binding, motor protein binding and endosomal transport (Gadadhar et al., 2017; Janke and Magiera, 2020; Sirajuddin et al., 2014) (Fig. 3B; Table S3).

The effect of polyglutamylation at the α- and β-tubulin C-terminal tails was recently studied in neurons (Fig. 3B). Deletion of the deglutamylase CCP1 induced tubulin hyperglutamylation, resulting in reduced LE and Lys anterograde trafficking (Bodakuntla et al., 2020). In accordance with these findings, CCP1 overexpression increased anterograde lysosomal trafficking (Zheng et al., 2022). Altered anterograde trafficking might be explained by changes in tau and MAP2 microtubule binding, given that both proteins affect kinesin-1 motility and their binding depends on tubulin polyglutamylation (Bonnet et al., 2001; Boucher et al., 1994). A peculiar tubulin PTM affecting kinesin-1-mediated endosomal transport is K40-acetylation (Fig. 3B). K40 is located in the microtubule lumen and cannot directly influence motor binding at the microtubule surface (Soppina et al., 2012). However, kinesin-1 prefers to bind to and move along K40-acetylated microtubules in cells, which enhances anterograde-directed transport (Reed et al., 2006; Dompierre et al., 2007; Cai et al., 2009; Guardia et al., 2016), although *in vitro* studies comparing microtubules polymerized from either isolated acetylated or deacetylated tubulin subunits could not detect a significant difference in kinesin-1 landing and velocity (Kaul et al., 2014; Walter et al., 2012). This provides an interesting *in vitro*–*in vivo* disparity and might indicate the involvement of additional factors, such as microtubule stability or MAP binding (Soppina et al., 2012). Finally, α-tubulin subunits undergo cycles of detyrosination and tyrosination at their exposed C-terminal tails, which is regulated by a currently unknown tubulin carboxy-peptidase and the tubulin tyrosine ligase TTL, respectively (Gadadhar et al., 2017) (Fig. 3B). Detyrosination supports kinesin-1 binding to microtubules, whereas kinesin-3 motors as well as the dynactin subunit p150^glued^ prefer binding to tyrosinated tubulin subunits (Guardia et al., 2016; McKenney et al., 2016; Kaul et al., 2014). Given that detyrosinated and tyrosinated microtubules are found at the perinuclear area and periphery, respectively, this PTM stimulates specific targeting of motor subtypes and endosomes to distinct cellular locations (Dunn et al., 2008; Guardia et al., 2016; Mohan et al., 2019).

The above described MAPs, tubulin isoforms and PTMs will likely act in conjunction when recruiting motor proteins to the microtubule. Therefore, studying solely the effects of single MAPs, tubulin isoforms or PTMs on endosomal transport is too minimalistic. It would be interesting to determine how the different factors cooperate in cells to regulate motor-endosome interactions and mobility, as this is still an open question.

## Proteins at ER–LE membrane contact sites regulate anterograde and retrograde transport

**Fig. 4.**
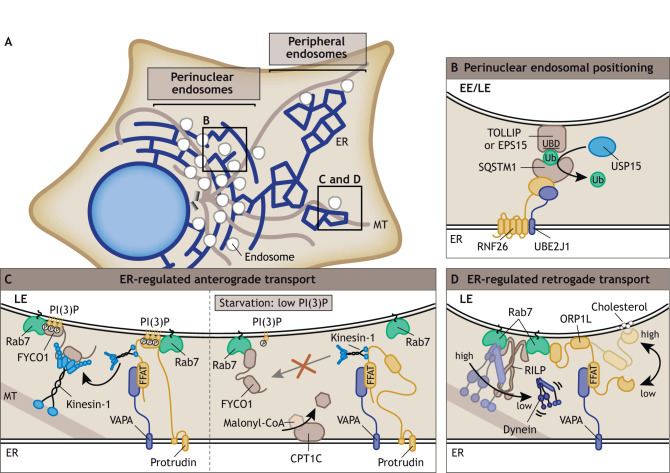
**Proteins at ER–LE contact sites regulate endosomal positioning and transport.** (A) Schematic overview of endosomal localization inside the cell, including the perinuclear endosomal cloud and the peripheral endosomes connected to the ER. (B) The ER-resident proteins RNF26 (an E3 ligase) and UBE2J1 (an E2 enzyme) recruit and ubiquitylate SQSTM1, which in turn binds the ubiquitin-binding endosomal proteins TOLLIP (LE/Lys) or EPS15 (EEs), linking endosomes to the ER membrane. UBD, ubiquitin-binding-domain; Ub, ubiquitin. (C) Left, the ER-embedded protein protrudin interacts with Rab7 and PI(3)P at the endosomal membrane and with VAPA at the ER membrane. Protrudin facilitates loading of kinesin-1 onto FYCO1–Rab7 at the endosomal membrane, promoting anterograde endosomal transport. Right, during starvation, kinesin-mediated transport is inhibited by low PI(3)P levels when FYCO1 and protrudin dissociate from the endosomal membrane. Malonyl-CoA synthesis is also inhibited, resulting in CPT1C that is not bound to malonyl-CoA, which prevents protrudin from transferring kinesin-1 to FYCO1. (D) Cholesterol (high) at the endosomal membrane interacts with the ORD domain of ORP1L, allowing dynein to interact with the RILP–Rab7 complex inducing retrograde endosomal transport. When endosomal cholesterol levels decrease (low), the ORP1L FFAT domain interacts with the ER-resident protein VAPA, leading to dissociation of dynein.

Endosomes are not stand-alone compartments, and in many cases their localization and transport are regulated by the ER. Endosomes interact with the ER through membrane contact sites (MCSs), which are <30 nm clefts between the ER and endosomal membranes that allow interactions between proteins residing in the opposing membranes. ER–LE contacts are especially prevalent as MCS formation appears to increase during endosome maturation (Friedman et al., 2013). The ER–LE contact sites enclose proteins regulating motor loading events, marking them as regulatory hubs for endosomal movement. Live-cell imaging experiments revealed that the endosomal localization in cells is characterized by two endosomal populations – a relatively immobile perinuclear one around the MTOC and a highly dynamic population at the cell periphery (Jongsma et al., 2016) (Fig. 4A). Both populations appear to be controlled by various ER-associated processes.

### The ER-resident E3 ligase RNF26 controls perinuclear endosomal positioning

At the perinuclear ER, contacts with endosomes are formed by the ER-resident E3 ubiquitin ligase RNF26. Together with its partnering E2 enzyme UBE2J1, RNF26 binds and ubiquitylates p62 (also known as SQSTM1), which in turn recruits the ubiquitin-binding endosomal adaptor proteins TOLLIP on LEs and EPS15 on EEs (Fig. 4B). This cascade bridges the endosomal population to the perinuclear ER, forming a stationary vesicular cloud (Cremer et al., 2021; Jongsma et al., 2016) and explains how the ER controls perinuclear endosome localization. Endosomes are released from the cloud by deubiquitylation events mediated by the deubiquitylating enzyme USP15 and subjected for transport into the periphery (Jongsma et al., 2016) (Fig. 4B). At the cell periphery, endosomes are highly dynamic and move bidirectionally along microtubule tracks while repeatedly contacting the ER membrane (Rocha et al., 2009).

### Protrudin controls anterograde endosomal transport at MCSs

One of the proteins present at ER–LE contact sites is protrudin, named after its role in protrusion formation (Shirane and Nakayama, 2006). Protrudin contains a transmembrane (TM) and a hairpin-domain inserted into the ER membrane and forms a bridge towards the endosome through interaction between its FYVE domain and PI(3)P, as well as an interaction with Rab7 at the endosomal membrane. The ER–LE connection is further stabilized by the protrudin FFAT motif, which interacts with the ER protein VAPA (Saita et al., 2009). At these contacts, protrudin recruits the kinesin-1 motor KIF5B and loads the motor onto the Rab7 effector FYCO1, thereby facilitating plus-end-directed LE movement (Matsuzaki et al., 2011; Raiborg et al., 2015) (Fig. 4C). Protrudin-controlled transport is affected by starvation. When amino acid levels are low, VPS34 activation is inhibited, which results in lower PI(3)P levels at the LE membrane (Hong et al., 2017; Byfield et al., 2005; Nobukuni et al., 2005). This leads to protrudin as well as FYCO1 dissociating from the LE membrane, followed by net retrograde transport and perinuclear LE clustering (Hong et al., 2017) (Fig. 4C). In addition, protrudin function is inhibited when low nutrient levels are sensed by the ER-localized nutrient sensor CPT1C. Under sufficient nutrient supply, CPT1C binds malonyl-CoA, thereby promoting protrudin-mediated anterograde LE transport (Palomo-Guerrero et al., 2019). However, during starvation, malonyl-CoA synthesis is inhibited and CPT1C that is not bound to malonyl-CoA prevents protrudin from transferring KIF5B to LEs (Palomo-Guerrero et al., 2019) (Fig. 4C).

### ORP1L controls retrograde endosomal transport at MCSs

Another protein present at ER–LE contact sites is the cholesterol sensor ORP1L, which affects dynein binding to the LE membrane. ORP1L associates to Rab7 without affecting Rab7 binding to its effector proteins RILP and FYCO1 (Ma et al., 2018; Tong et al., 2019; Johansson et al., 2007). ORP1L can adopt different conformations depending on the cholesterol levels at the endosomal membrane. High cholesterol levels allow the C-terminal ORD domain of ORP1L to interact with cholesterol at the LE membrane, preventing its FFAT motif from binding to VAPA at the ER membrane (Rocha et al., 2009). Instead, ORP1L facilitates RILP binding to p150^glued^, allowing dynein-mediated LE transport towards the perinuclear area (Rocha et al., 2009) (Fig. 4D). When cholesterol levels are low, the ORD domain is released from the LE membrane, which induces a conformational change that allows the FFAT motif to interact with VAPA. This leads to p150^glued^ dissociating from RILP, releasing the dynein motor and resulting in subsequent peripheral LE localization when kinesin motors take over (Rocha et al., 2009; van der Kant et al., 2013) (Fig. 4D). This mechanism adds cholesterol content as an additional factor in control of LE mobility as sensed by ORP1L and mediated by the ER.

As discussed above, dynein and kinesin motor recruitment to the endosomal membrane is regulated by ER-membrane-embedded molecules, including the proteins protrudin and ORP1L, as well as cholesterol and phospholipids on the LE and Lys membrane (Rocha et al., 2009; Raiborg et al., 2016). Whether the different mechanisms, such as kinesin loading and dynein dissociation, work in concert within the same MCS to control bidirectional LE transport is still unclear.

## Conclusions and perspectives

Endosomal movement was originally considered simple, with a motor protein binding to the endosome resulting in its transport, yet decades of studies on endosomal transport have revealed a far more complex situation with every discovery opening many more layers of control. The diversity in motor anchors at the endosomal membrane as well as MAPs and tubulin-PTMs affecting motor recruitment to the microtubule add to the complexity of endosomal transport regulation. In addition, different endosomal locations have different control systems – the ER protein RNF26 controls perinuclear endosomal location whereas other mechanisms, including ORP1L and protrudin-mediated motor loading, regulate fast moving peripheral endosomes. While many components of the endosomal transport machinery have been identified, we may still miss essential factors, owing to limitations of the current experimental approaches. Another consequence of the wide number of components in control of transport is that it becomes virtually impossible to study all of these simultaneously in isolation. Studying endosomal transport in the context of a living cell, using GFP-knock-in systems, optogenetics and chemical biology combined with time-lapse microscopy, may allow further understanding of the complex and fascinating endosomal dance and the many contributing partners.

## Supplementary Material

10.1242/joces.259689_sup1 Supplementary information
