# Supplementary material for: Choreographing the motor-driven endosomal dance
Source: J Cell Sci. 2022 Nov 16;136(5):jcs259689. doi: 10.1242/jcs.259689 (PMC9845747; doi:10.1242/jcs.259689)
Supplement: Supplementary information [file joces-136-259689-s1.pdf]

Table S1. Examples of protein complexes attaching molecular motors to endosomes, relates to Fig. 1

Cargo	Motor	Mechanism	References
EE	Dynein-dynactin (DLIC-1)	Rab4	(Bielli et al., 2001)
EE	Kinesin-2 (KIF3A/B)	Rab4-unkown	(Dey et al., 2017)
EE	Kinesin-3 (KIF16B)	Rab5-VPS34-PI(3)P	(Christoforidis et al., 1999, Hoepfner et al., 2005)
EE	Dynein-dynactin (DLIC1) and kinesin-3 (KIF1C)	Rab5-FHF complex (FHIP1B/Hook1-3/FTS)	(Bielska et al., 2014, Christensen et al., 2021, Guo et al., 2016, Kendrick et al., 2019, Schroeder and Vale, 2016, Yao et al., 2014, Zhang et al., 2014)
SE	kinesin-2 (KIF3B)	Rab11-Rip11/FIP5	(Schonteich et al., 2008)
SE	Kinesin-3 (KIF13A)	Rab10/Rab11/Rab22A-BLOC1/2	(Patel et al., 2021, Shakya et al., 2018, Delevoye et al., 2014, Etoh and Fukuda, 2019)
SE	Dynein-Dynactin (DLC1)	PI(3)P-SNX4-KIBRA	(Skanland et al., 2009, Traer et al., 2007)
SE	Dynein-Dynactin (DLIC1)	Rab11-FIP3	(Horgan et al., 2010)
LE/Lys	Dynein-dynactin ($p150^{glued}$/DLIC1)	Rab7-RILP-ORP1L-Cholesterol	(Johansson et al., 2007, Jordens et al., 2001)
LE/Lys	Kinesin-1 (KIF5B)	Rab7-FYCO1-PI(3)P	(Pankiv et al., 2010, Raiborg et al., 2015)
LE/Lys	Kinesin-1 (KIF5B-KLC)	BORC-Arl8b-SKIP	(Guardia et al., 2016, Pu et al., 2015, Rosa-Ferreira and Munro, 2011)
LE/Lys	Kinesin-3 (KIF1A/B-CC3)	BORC-Arl8b	(Guardia et al., 2016, Pu et al., 2015)
LE/Lys	Dynein-dynactin	Arl8b-RUFY3/4-JIP4	(Keren-Kaplan et al., 2022, Kumar et al., 2022)
Lys	Kinesin-1 Dynein-dynactin	LRRK2-Rab35-Rab10-JIP4	(Bonet-Ponce et al., 2020)
Lys	Dynein-Dynactin	$PI(3,5)P_2$-TRPML1-ALG2	(Li et al., 2016)

Journal of Cell Science • Supplementary information

Table S2. The influence of MAPs on motor proteins, relates to Fig. 3

	**Stimulated**	**Not affected**	**Inhibited**
MAP2		Kinesin-3 (KIF1)<br>(Gumy et al., 2017)	Kinesin-1 (KIF5B)<br>(Monroy et al., 2020, Gumy et al., 2017, Hagiwara et al., 1994)<br>Kinesin-3 (KIF1A)<br>(Monroy et al., 2020)<br>Dynein-dynactin<br>(Paschal et al., 1989)
Tau		Dynein-dynactin-BICD<br>(Chaudhary et al., 2018, Monroy et al., 2018)<br>Kinesin-2 (KIF3A)<br>(Hoeprich et al., 2014, Chaudhary et al., 2018)	Kinesin-1 (KIF5B)<br>(Monroy et al., 2020, Chaudhary et al., 2018)<br>Kinesin-3 (KIF1A)<br>(Monroy et al., 2018, Monroy et al., 2020)
MAP4	Kinesin-2 (KIF3A)<br>(Semenova et al., 2014)		Kinesin-1<br>(Tokuraku et al., 2007)<br>Dynein-dynactin<br>(Semenova et al., 2014)
MAP7	Kinesin-1 (KIF5B)<br>(Hooikaas et al., 2019, Monroy et al., 2018, Chaudhary et al., 2019)	Dynein-dynactin-BICD<br>(Monroy et al., 2018)<br>Kinesin-2<br>(Chaudhary et al., 2019)	Kinesin-3 (KIF1A)<br>(Monroy et al., 2020, Monroy et al., 2018)<br>Dynein-dynactin-BICD<br>(Ferro et al., 2022)
MAP7D1	Kinesin-1 (KIF5B)<br>(Hooikaas et al., 2019, Song et al., 2013)		
MAP7D2	Kinesin-1 (KIF5A/B/C)<br>(Pan et al., 2019)		
MAP7D3	Kinesin-1 (KIF5B)<br>(Hooikaas et al., 2019)		
MAP9	Kinesin-3 (KIF1A)<br>(Monroy et al., 2020)		Kinesin-1 (KIF5B)<br>(Monroy et al., 2020)<br>Dynein-dynactin-BICD<br>(Monroy et al., 2020)

Journal of Cell Science • Supplementary information

Table S3. The effects of tubulin PTM on motor proteins, relates to Fig. 3

**Modification**		**Affected**	**Not affected**
Acetylation<br>(α-tubulin)	Addition of an acetyl group to K40	Kinesin-1 (KIF5B)<br>(Guardia et al., 2016, Reed et al., 2006, Cai et al., 2009)	Kinesin-2 (KIF17)<br>(Cai et al., 2009)<br>Kinesin-3 (KIF1A)<br>(Cai et al., 2009)
Tyrosination<br>(α-tubulin)	Presence of tyrosine at the C-terminal tail	Kinesin-3 (KIF1A/B)<br>(Guardia et al., 2016)<br>Dynein-dynactin ($p150^{glued}$)<br>(Honnappa et al., 2006, McKenney et al., 2016, Mishima et al., 2007)<br>Kinesin-13<br>(Sirajuddin et al., 2014)	Kinesin-2<br>(Sirajuddin et al., 2014)
De-tyrosination<br>(α-tubulin)	Removal of C-terminal tyrosine	Kinesin-1 (KIF5)<br>(Cai et al., 2009, Kaul et al., 2014, Liao and Gundersen, 1998, Mohan et al., 2019, Reed et al., 2006)<br>Kinesin-2<br>(Sirajuddin et al., 2014)<br>Kinesin-7 (CENP-E)<br>(Barisic et al., 2015)	Kinesin-2 (KIF17)<br>(Cai et al., 2009)<br>Kinesin-3 (KIF1A)<br>(Cai et al., 2009)
Polyglutamylation<br>(α- and β-tubulin)	Addition of multiple glutamate residues at the C-terminal tail	Kinesin-1 (KIF5)<br>(Sirajuddin et al., 2014)<br>Kinesin-2 (KIF3A)<br>(Sirajuddin et al., 2014)<br>Kinesin-3 (KIF1A)<br>(Ikegami et al., 2007, Lessard et al., 2019)	Kinesin-2 (KIF3A)<br>(Ikegami et al., 2007)<br>Kinesin-1 (KIF5)<br>(Ikegami et al., 2007)<br>Kinesin-13 (Sirajuddin et al., 2014)<br>Dynein-dynactin<br>(Sirajuddin et al., 2014)
Polyglycylation<br>(α- and β-tubulin)	Addition of glycine residues at the C-terminal tail	Microtubule stabilization<br>(Bosch Grau et al., 2013, Rogowski et al., 2009)	
Polyamination<br>(β-tubulin)	Addition of an amine group to Q15 of β-tubulin	Microtubule stabilization<br>(Song et al., 2013)	
O-GlcNAcylation<br>(α- and β-tubulin)	Addition of a sugar-chain to α- and β-tubulin	Microtubule dynamics<br>(inhibits microtubule polymerization)<br>(Ji et al., 2011, Walgren et al., 2003)	
Ubiquitination<br>(α-tubulin)	Addition of a ubiquitin moiety	Microtubule stabilization<br>(Mukherjee et al., 2017)	

Journal of Cell Science • Supplementary information

BARISIC, M., SILVA E SOUSA, R., TRIPATHY, S. K., MAGIERA, M. M., ZAYTSEV, A. V., PEREIRA, A. L., JANKE, C., GRISHCHUK, E. L. & MAIATO, H. 2015. Mitosis. Microtubule detyrosination guides chromosomes during mitosis. *Science,* 348**,** 799-803.

GUMY, L. F., KATRUKHA, E. A., GRIGORIEV, I., JAARSMA, D., KAPITEIN, L. C., AKHMANOVA, A. & HOOGENRAAD, C. C. 2017. MAP2 Defines a Pre-axonal Filtering Zone to Regulate KIF1-versus KIF5-Dependent Cargo Transport in Sensory Neurons. *Neuron,* 94**,** 347-362 e7.

HOEPRICH, G. J., THOMPSON, A. R., MCVICKER, D. P., HANCOCK, W. O. & BERGER, C. L. 2014. Kinesin's neck-linker determines its ability to navigate obstacles on the microtubule surface. *Biophys J,* 106**,** 1691-700.

HONNAPPA, S., OKHRIMENKO, O., JAUSSI, R., JAWHARI, H., JELESAROV, I., WINKLER, F. K. & STEINMETZ, M. O. 2006. Key interaction modes of dynamic +TIP networks. *Mol Cell,* 23**,** 663-71.

IKEGAMI, K., HEIER, R. L., TARUISHI, M., TAKAGI, H., MUKAI, M., SHIMMA, S., TAIRA, S., HATANAKA, K., MORONE, N., YAO, I., CAMPBELL, P. K., YUASA, S., JANKE, C., MACGREGOR, G. R. & SETOU, M. 2007. Loss of alpha-tubulin polyglutamylation in ROSA22 mice is associated with abnormal targeting of KIF1A and modulated synaptic function. *Proc Natl Acad Sci U S A,* 104**,** 3213-8.

LESSARD, D. V., ZINDER, O. J., HOTTA, T., VERHEY, K. J., OHI, R. & BERGER, C. L. 2019. Polyglutamylation of tubulin's C-terminal tail controls pausing and motility of kinesin-3 family member KIF1A. *J Biol Chem,* 294**,** 6353-6363.

LIAO, G. & GUNDERSEN, G. G. 1998. Kinesin is a candidate for cross-bridging microtubules and intermediate filaments. Selective binding of kinesin to detyrosinated tubulin and vimentin. *J Biol Chem,* 273**,** 9797-803.

MISHIMA, M., MAESAKI, R., KASA, M., WATANABE, T., FUKATA, M., KAIBUCHI, K. & HAKOSHIMA, T. 2007. Structural basis for tubulin recognition by cytoplasmic linker protein 170 and its autoinhibition. *Proc Natl Acad Sci U S A,* 104**,** 10346-51.

PAN, X., CAO, Y., STUCCHI, R., HOOIKAAS, P. J., PORTEGIES, S., WILL, L., MARTIN, M., AKHMANOVA, A., HARTERINK, M. & HOOGENRAAD, C. C. 2019. MAP7D2 Localizes to the Proximal Axon and Locally Promotes Kinesin-1-Mediated Cargo Transport into the Axon. *Cell Rep,* 26**,** 1988-1999 e6.

ROGOWSKI, K., JUGE, F., VAN DIJK, J., WLOGA, D., STRUB, J. M., LEVILLIERS, N., THOMAS, D., BRE, M. H., VAN DORSSELAER, A., GAERTIG, J. & JANKE, C. 2009. Evolutionary divergence of enzymatic mechanisms for posttranslational polyglycylation. *Cell,* 137**,** 1076-87.

TOKURAKU, K., NOGUCHI, T. Q., NISHIE, M., MATSUSHIMA, K. & KOTANI, S. 2007. An isoform of microtubule-associated protein 4 inhibits kinesin-driven microtubule gliding. *J Biochem,* 141**,** 585-91.

WALGREN, J. L., VINCENT, T. S., SCHEY, K. L. & BUSE, M. G. 2003. High glucose and insulin promote O-GlcNAc modification of proteins, including alpha-tubulin. *Am J Physiol Endocrinol Metab,* 284**,** E424-34.

Journal of Cell Science • Supplementary information
